# Identification of gene expression biomarkers to predict clinical response to methotrexate in patients with rheumatoid arthritis

**DOI:** 10.1007/s10067-023-06814-2

**Published:** 2023-11-17

**Authors:** Andriko Palmowski, Cindy Strehl, Moritz Pfeiffenberger, Thomas Häupl, Martina Schad, Jim Kallarackal, Ulrich Prothmann, Denise Dammann, Mark Bonin, Andreas Brandt, Udo Schneider, Timo Gaber, Frank Buttgereit

**Affiliations:** 1 Department of Rheumatology and Clinical Immunology, Charité - Universitätsmedizin Berlin, Corporate Member of Freie Universität Berlin and Humboldt-Universität Zu Berlin, 10117 Berlin, Germany; 2 Section for Biostatistics and Evidence-Based Research, The Parker Institute, Bispebjerg and Frederiksberg Hospital, Copenhagen, Denmark; 3 German Rheumatism Research Centre (DRFZ) Berlin, Institute of the Leibniz Association, 10117 Berlin, Germany; 4 OakLabs Bio Inc, Raleigh, NC USA; 5 Klinik Püttlingen - Knappschaftsklinikum Saar, Püttlingen, Germany; 6 medac Gesellschaft Für Klinische Spezialpräparate mbH, Wedel, Germany

**Keywords:** Rheumatoid arthritis, Methotrexate, Response, Prediction, Biomarkers, Gene signature, Pharmacogenomics

## Abstract

**Objectives:**

To identify biomarkers at the gene expression level to predict response to methotrexate (MTX) in patients with rheumatoid arthritis (RA).

**Methods:**

MTX-naïve patients with RA were started on MTX and followed up over three months. The disease activity score 28 (DAS28) was used to classify patients into responders and non-responders. Genome-wide gene expression analysis was performed in CD4 + and CD14 + mononuclear cells sampled from whole blood at baseline to identify differentially expressed genes in responders versus non-responders. Gene selection methods and prediction modelling obtained the most relevant differentially expressed genes. A logistic regression prediction model was subsequently constructed and validated via bootstrapping. The area under the receiver operating characteristic (AUC) curve was calculated to judge model quality.

**Results:**

Seventy-nine patients with RA (53.4 ± 13.9 years, 74.7% females) were enrolled, and 70 finished the study with a documented treatment EULAR response (77.1% responders). Forty-six differentially expressed genes were found. The most promising genes were KRTAP4-11, LOC101927584, and PECAM1 in CD4 + cells and PSMD5 and ID1 in CD14 + cells. The final prediction model using these genes reached an AUC of 90%; the validation set’s AUC was 82%.

**Conclusions:**

Our prediction model constructed via genome-wide gene expression analysis in CD4 + and CD14 + mononuclear cells yielded excellent predictions. Our findings necessitate confirmation in other cohorts of MTX-naïve RA patients. Especially if used in conjunction with previously identified clinical and laboratory (bio)markers, our results could help predict response to MTX in RA to guide treatment decisions.

**Table:** 

**Key Points** • *Patients with rheumatoid arthritis may or may not respond to treatment with methotrexate, which is the recommended first-line drug in guidelines around the world.* • *In non-responders, valuable time is lost until second-line treatments are started.* • *This study aimed at predicting response to methotrexate by identifying differentially expressed genes from peripheral blood samples.* • *The final prediction model yielded excellent prognostic values, but validation in other cohorts is necessary to corroborate these findings.*

**Supplementary Information:**

The online version contains supplementary material available at 10.1007/s10067-023-06814-2.

## Introduction

Methotrexate (MTX) has been one of the most important drugs for the treatment of rheumatoid arthritis (RA) for more than 30 years [1]. The European League Against Rheumatism recommendations suggest it as a first-line treatment and call it an “anchor drug” [2], similarly to the American College of Rheumatology guidelines [3]. As a consequence, MTX is very widely used. In Germany, for example, 57% of patients with RA in a recent real-world study used MTX [4].

Unfortunately, not all patients respond adequately to MTX. Some patients have high disease activity with consequences such as joint destruction or systemic manifestations. In these patients, valuable time is lost until non-response is documented. An earlier switch to a different DMARD could quickly alleviate symptom burden and preserve joint quality. Therefore, it would be precious to predict successful MTX response as patients with a low likelihood of response to MTX could directly start another DMARD.

Prior studies have investigated clinical, nongenetic, and genetic biomarkers of response [5], but no methods have yet found their way into daily practice. This is probably partly due to the complexity and cost of biomarker assessment but also due to insufficient predictive quality.

Monocytes play a central role in the initiation of inflammation in RA. High numbers in the peripheral blood are associated with RA disease activity [6] and have been shown to predict poorer responses to MTX, but these findings have not been validated to date [7]. CD3 + /CD4 + T cells are also thought to be major players in the pathogenesis of RA. CD4 + T cells are, e.g., enriched in affected joints of RA patients, and expanded CD4 + T cell clones were identified in the synovium of RA patients [8]. Additionally, both CD14 + monocytes and CD3 + /CD4 + T-cells have the advantage of being available from peripheral blood easily and in sufficient numbers to allow for gene expression analyses.

Our objective was to assess CD3 + /CD4 + T-cells and CD14 + monocytes at the gene expression level to predict response to MTX in order to personalize the treatment approach and ultimately improve outcomes in RA.

## Material and methods

### Study design and participants

This was an observational cohort study. Male and female patients aged 18 years or older with a documented history of RA according to the criteria of the American College of Rheumatology [9] were eligible for this study, whose enrolment period lay between May 2014 and September 2016. Patients were required to be MTX-naïve. Enrolled patients were started on subcutaneous MTX (scMTX), 15 mg/week. This dose was held constant. The subcutaneous formulation was used to ensure adequate drug uptake, reduce intolerance as much as possible, and optimise adherence. The decision to start scMTX was at the discretion of each physician, following standard treatment guidelines and recommendations. Patients were using glucocorticoids (prednisone 5 mg/day). Intra-articular injection histories were not questioned. Exclusion criteria included pregnancy or breastfeeding (in women), history of malignancy or anaemia (haemoglobin concentration ≤ 10.0 g/dl), opiate intake, alcohol or drug abuse, inability to provide informed consent, and severe psychiatric comorbidities. The study PreTheraX and the study protocol was approved by the ethics committees (April 15, 2014; EA1/073/14) and institutional review boards. All patients provided written informed consent. No sample size calculations were performed beforehand as this was considered a pilot study.

### Study procedures

At the baseline and second visit after 3 months of scMTX treatment, eligible participants were physically examined, and standard blood and urine tests were performed. This included measuring erythrocyte sedimentation rates (ESR) and C-reactive protein (CRP). At both time points, participants rated pain and stiffness using a visual analogue scale (VAS). The disease activity score of 28 joints (DAS28) was calculated based on four variables: 28-tender joint count (TJC28), 28-swollen joint count (SJC28), ESR or CRP, and VAS. Three months after the start of scMTX, Patients were individually classified as non-, moderate, or good responders according to EULAR recommendations [10]. The 3-month time span was chosen as the EULAR recommendations advise to assess patients for improvement after 3 months [11].

### Sample preparation

Peripheral blood samples (40 ml) were collected in heparinised tubes at the screening visit (i.e., before the start of scMTX). This blood sampling was performed during the opening hours of the outpatient clinic or in the morning in case of inpatient stay. Immediately after retrieval of the blood samples, peripheral blood mononuclear cells (PBMC) were isolated using density gradient centrifugation. PBMC were resuspended in 0.5% bovine serum albumin (Sigma-Aldrich) in phosphate-buffered saline (PBS/BSA; pH 7.4) and split into two falcon tubes. After blocking the unspecific binding with 10% (v/v) Flebogamma on ice for five minutes, 1/3 of the PBMC were labelled with anti‐human‐CD4‐MicroBeads, whereas 2/3 were labelled with anti-human‐CD14‐MicroBeads. According to the manufacturer’s instructions, the corresponding immune cell types were enriched by MACS sort (Miltenyi Biotec GmbH). Cell count was determined using a Neubauer counting chamber. Cell purity was analysed by labelling with appropriate monoclonal antibodies (CD3 [clone UCHT1] and CD4 [clone TT1] for T cells; CD14 [clone TM1] for monocytes). All antibodies were distributed by the core facility of the Deutsches Rheumaforschungszentrum. Cells with more than 90% purity, as analysed by flow cytometry, were stored at − 80 °C until further processing.

### RNA isolation, quality control, and hybridization

Total RNA was isolated using the RNeasy Mini Kit (Qiagen) with on-column DNase digestion with the RNAse-free DNase Set (Qiagen). All RNA samples underwent quality control to determine RNA integrity numbers (RIN) using the RNA 6000 Pico Kit with the 2100 Bioanalyzer (Agilent Technologies) and the Nanodrop 2000 spectrophotometer (Thermo Scientific). All samples had a RIN value above 8.

The Low Input QuickAmp Labeling Kit (Agilent Technologies) was used. It started with the 1st strand synthesis using oligo-dT primer, followed by the 2nd strand synthesis. Afterwards, fluorescent cRNA (complementary RNA) is generated in an in vitro transcription with cyanine 3-CTP.

The microarray was used with the design ID 039494 (Agilent Technologies) for genome-wide gene expression analysis. And 600 ng of each cRNA was hybridised on those 8 × 60 K microarrays at 65 °C for 17 h using the Gene Expression Hybridization Kit (Agilent Technologies) and Agilent’s recommended hybridisation chamber and oven. Next, microarrays were washed with Agilent buffers, once with the Gene Expression Wash Buffer 1 for one minute at ambient temperature, followed by a second wash with preheated (37 °C) Gene Expression Wash Buffer 2 for 1 min. Fluorescence signals on microarrays were detected by the SureScan Microarray Scanner (Agilent Technologies) at a resolution of 3 microns for SurePrint G3 Gene Expression Microarrays, generating a 20-bit TIFF file. Agilent’s Feature Extraction software version 11 was used to read and process the TIFF files.

### Analysis of microarray data

Raw data were analysed using DirectArray Software (OakLabs, Hennigsdorf, Germany). The signal distributions of the raw data were visualised using box plots to identify potential issues for individual samples. Samples were quantile normalised and subjected to statistical analysis by applying Welch’s test and calculating log2 fold changes for each gene. *P* < 0.050 was considered statistically significant.

### Validation via quantitative PCR

Total RNA was isolated at OakLabs GmbH. TaqMan® Reverse Transcription Reagents Kit (Thermo Fisher, USA) was used for cDNA synthesis with more than 50 ng per reaction. In contrast, Sensiscript Reverse Transcriptase Kit (Qiagen, Germany) was used for cDNA synthesis with less than 50 ng per reaction. Primers were designed using Primer Blast (NCBI, USA). Probes (6FAM as reporter dye, BBQ as a quencher) were created using the designing tool IDT DNA (Integrated DNA Technologies, USA). Both, primer and probes were synthesised by TIB MolBiol (Berlin, Germany). Sequences are summarised in Supplementary Table 2. Quantitative PCR (qPCR) was performed in the Stratagene Mx3000PTM (Agilent Technologies Inc., USA) using the Brilliant III Ultra-Fast QPCR Master Mix (Agilent Technologies, USA) according to the manufacturer’s instructions with the following temperature profile: 3 min of initial denaturation at 95 °C and 50 cycles of 20 s at 95 °C and 20 s at 60 °C. All values were generated in duplicate and were corrected concerning efficiency.

### Statistical analysis

First, study participants’ baseline demographics and disease characteristics were descriptively analysed and presented both for the overall cohort and stratified by MTX response. Normality was assessed using the D’Agostino-Pearson test on the group of study completers. For comparability, data from all other groups were presented accordingly. Normally distributed data were presented as mean and standard deviation and non-normally distributed data as median and interquartile percentiles.

The entire data set has been separated into a discovery and validation data set. The size of the validation data set consisted of 40% of the total samples. We used a logistic regression model to identify the most promising genes as a logistic regression model requires only very limited computing resources. We used a support vector machine to combine predictions based on CD4 + and CD14 + genes for the final model. We used a bootstrap process to compute reliable statistical metrics and errors.

We calculated sensitivity, specificity, positive predictive values, accuracy, and the area under the curve of the receiver-operating characteristic (AUC ROC). The AUC ROC is used to rate the prediction quality, providing values between 0 and 1. A value of 1 denotes a perfect prediction model.

Spearman’s correlation coefficients were used to validate quantitative PCR to determine the correlation between microarray raw data (light intensity) and the amount of PCR product in fg.

## Results

### Study population

**Fig. 1:**
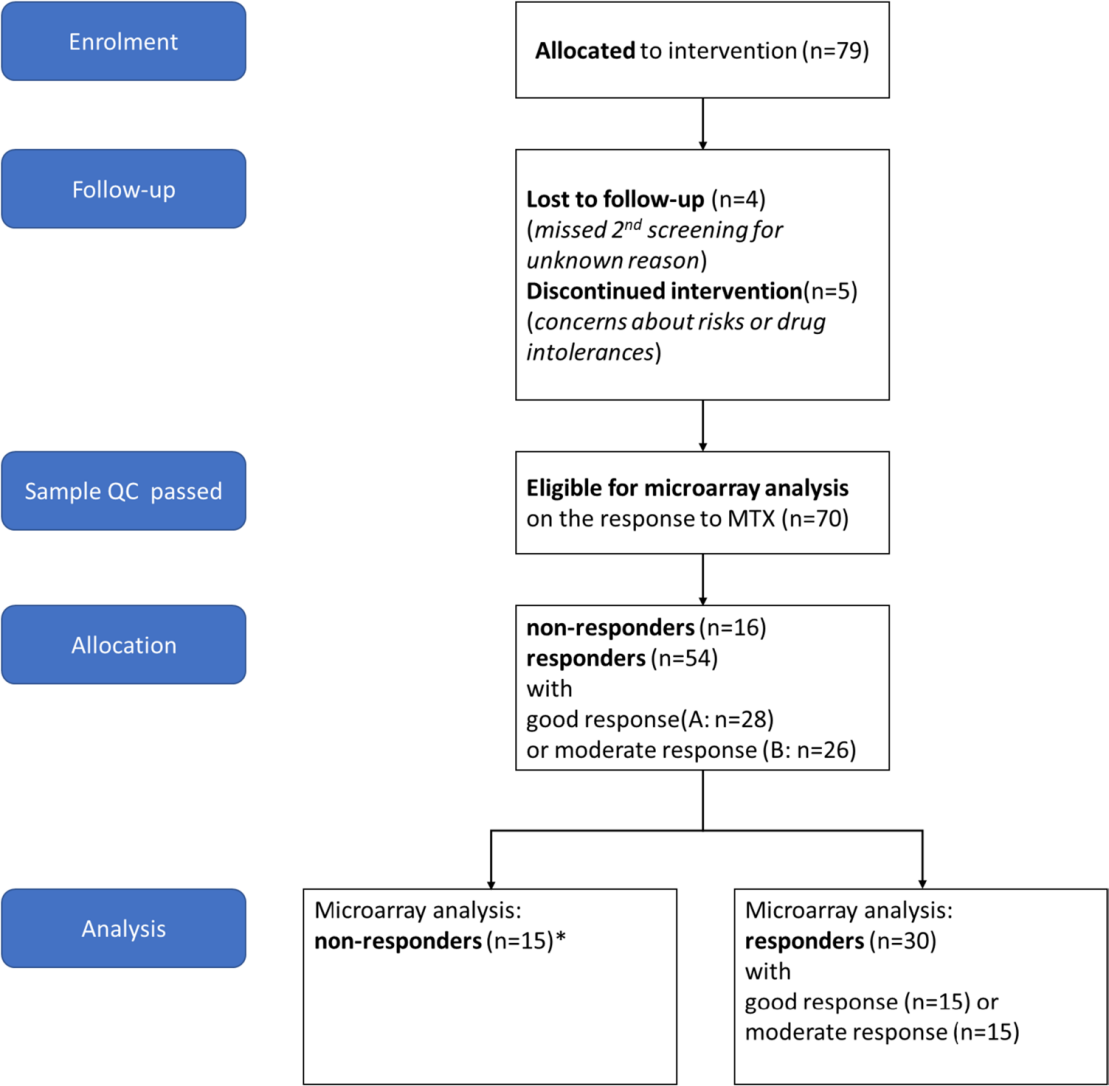
Patient enrolment. Seventy-nine patients were enrolled in this study. *One specimen was analysed via Microarray but excluded because the patient switched from scMTX to oral MTX. MTX, methotrexate

The characteristics of all RA patients enrolled in the PreTheraX study (*n* = 79) and those finishing the study (*n* = 70) are summarised in Table 1. The predominantly female study participants (74.7%) were aged 53.7 years (range 22–89). Seventy patients (88.6%) completed the study (Fig. 1). The main reason for early withdrawal was the discontinuation of MTX due to patient concerns or drug intolerance. According to the EULAR response criteria, 16 patients (22.8%) were classified as non-responders, whereas 54 (77.1%) were classified as responders: 28 showed a good response, and 26 showed a moderate response. Demographics were generally well-balanced between the response groups.

**Table 1:** Demographics and disease characteristics at baseline visit and disease characteristics after 3 months of scMTX treatment. Data are mean (SD), median (interquartile range), or number (%). *Partially not specified. **One missing value (doctor’s assessment). *NA* not available

	Demographics and disease characteristics	RA patients enrolled *n* = 79	Study completers *n* = 69	Non-responders *n* = 15	Responders *n* = 54	Good response *n* = 28	Moderate response *n* = 26
Baseline	Age (years)	53.4 ± 13.9	52.2 ± 13.4	58.0 ± 10.8	50.3 ± 13.7	49.2 ± 14.7	51.8 ± 12.5
	Female sex	59 (74.7%)	50 (72.5%)	9 (56.3%)	42 (77.8%)	21 (75%)	21 (80.8%)
	BMI	24.5 (20.1, 30.2)	25.5 (22.5, 29.1)	27.1 (24.6, 29.1)	25.6 (21.7, 29.9)	25.2 (21.2, 29.3)	24.6 (22.5, 28.5)
	Smoker	26/58* (44.8%)	26/56* (46.4%)	7/14* (50%)	19/41* (46.3%)	9/21* (42.9%)	10/20* (50%)
	RF positive	27/63* (42.9%)	34/60* (56.7%)	8 (50%)	26/44* (59.1%)	16/24* (66.7%)	10/20* (50%)
	ANA positive	36/55* (65.5%)	32/52* (61.5%)	9/14* (64.3%)	24/38* (63.2%)	12/22* (54.5%)	12/16* (75%)
	ACPA positive	39/62* (62.9%)	37/59* (62.7%)	10/14* (71.4%)	27/44* (61.4%)	15/24* (62.5%)	12/20* (60%)
	Glucocorticoids before MTX treatment	23/64* (35.9%)	23/61* (37.7%)	10 (62.5%)	13/45* (28.9%)	4/24* (16.7%)	10/20* (50%)
	NSAR before MTX treatment	32/64* (50%)	31/61* (50.8%)	5 (33.3%)	26/45* (57.8%)	14/24* (58.3%)	12/21* (57.1%)
	DAS 28	5.1 ± 1.2	5.1 ± 1.2	4.5 ± 1.1	5.3 ± 1.2	5.3 ± 1.1	5.3 ± 1.2
3 Months	Tender joint count (TJC28)	-	0.5 (0.0, 3.0)	3.0 (0.0, 10.0)	0.0 (0.0, 2.0)	0.0 (0.0, 0.0)	2.0 (1.0, 6.0)
	Swollen joint count (SJC28)	-	1.0 (0.0, 2.0)	1.5 (0.3, 2.8)	0.5 (0.0, 2.0)	0.0 (0.0, 1.0)	2.0 (1.0, 4.0)
	ESR (mm/h)	-	17.0 (8.3, 30.0)	26.0 (20.0, 62.0)	14.0 (8.0, 28.0)	12.5 (7.5, 26.5)	16.0 (8.0, 30.0)
	CRP (mg/dl)	-	2.8 (1.0, 5.5)	1.8 (1.0, 8.0)	2.9 (0.9, 4.7)	2.0 (0.9, 3.5)	3.9 (1.2, 7.0)
	Pain (VAS; 0–100)	-	20.0 (10.0, 42.8)	42.0 (30.0, 70.0)	20.0 (5.0, 30.0)	10.0 (0.0, 20.0)	20.0 (20.0, 46.3)
	DAS 28	-	3.2 ± 1.3**	4.6 ± 1.2	2.8 ± 1.1**	2.1 ± 0.6	3.7 ± 0.8**
	DAS improvement	-	1.9 ± 1.6**	− 0.1 ± 0.5	2.5 ± 1.3**	3.2 ± 1.1	1.6 ± 0.8**

### Microarray analysis: extraction of a robust gene signature

A genome-wide gene expression analysis of CD14 + and CD4 + samples from 45 patients (15 non-responders; 15 moderate responders; 15 good responders) was performed. Significantly differentially expressed genes were detected in 10 good responders and 10 non-responders for both CD14 + and CD4 + samples. A principal component analysis (PCA) was performed for these genes on specimens of all patients. A preliminary gene signature was found for the two cell types, CD4 + and CD14 + , with a sensitivity of 0.90 ± 0.11 and specificity of 0.74 ± 0.13. A summary of the identified 46 genes is given in Supplementary Table 1.

### Microarray analysis: restriction to a smaller gene signature

Using the 46 genes of the previously introduced robust gene signature as a starting point, we strove to reduce the number of genes further to obtain our final prediction model. We used gene selection methods to reduce the number of 3/2 most relevant genes for the CD4 + /CD14 + cell type. Then, we used a bootstrap resampling method (disregarding, in this case, the condition of age insignificance between the responder and non-responder sets) which in each iteration separately respected the independence of training and validation set samples. The obtained gene signatures that resulted in the best in-average AUC ROC score over the bootstrap samples for the two considered cell types are shown in Table 2.

**Table 2:** Final gene signatures employed to construct the final prediction model for the two cell types

Cell type	Array position	Gene ID	Gene symbol	Gene name
CD4 +	A_23_P4400	NM_033059	KRTAP4-11	Keratin-associated protein 4–11
	A_21_P0006871	XR_246208	LOC101927584	Uncharacterized
	A_33_P3229402	NM_000442	PECAM1	Platelet/endothelial cell adhesion molecule 1
CD14 +	A_33_P3305243	ENST00000373903	PSMD5	Proteasome (prosome, macropain) 26S subunit, non-ATPase, 5
	A_23_P252306	NM_002165	ID1	Inhibitor of DNA binding 1, dominant negative helix-loop-helix protein

The final prediction model was obtained by combining the models for the two cell types into one model using the probability scores of the two models as the input features of a support vector machine model that was fit to employ the same resampling technique again.

**Fig. 2:**
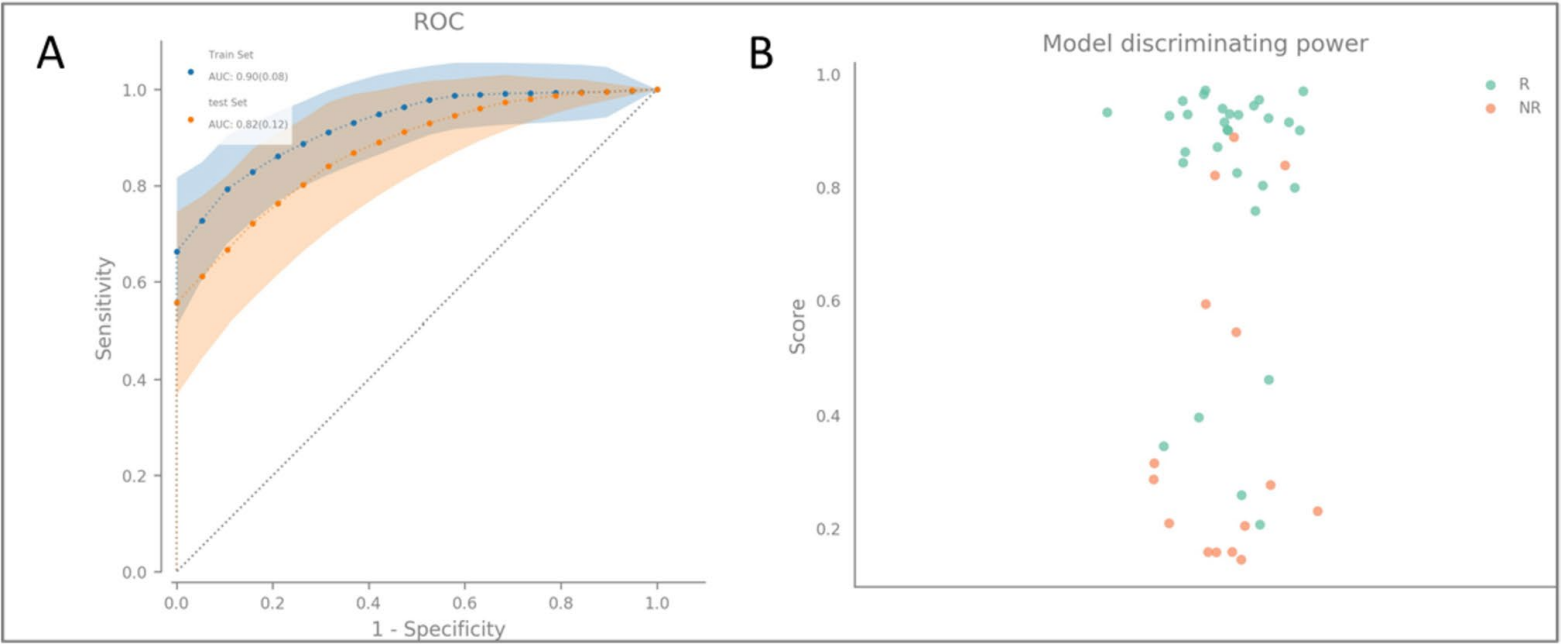
**A** In-average ROC of the final prediction model for the training and validation data in the applied resampling method. **B** Strip plot showing the classification scores of the prediction model with the prediction score closest to the resampling mean score applied to all available samples. R, responder; NR, non-responder

Its performance is demonstrated in Fig. 2A. It shows the average ROC and associated error levels of the predictions within the training and validation portions of samples throughout the bootstrap resampling. As can be seen, the train and validation curves overlap over the whole range of accurate positive rates.

To estimate in-average ROC curves with associated error bands, we first binned the false-positive rates as evaluated in the resampling into equally sized bins and accumulated the valid positive rates within each container. We then computed the per-bin mean values and standard deviations combined into a single curve using a linear interpolation, which shows smooth ROC curves.

Note that the chosen resampling-based analysis strategy, which is not relying on a single split between train and test data, goes in line with the adopted strategy explained in the paragraph “extraction of a robust gene signature.” Moreover, the resampling technique assures obtaining robust results considering the low number of samples.

The discriminating power of our prediction model is shown by combining the prediction scores into a strip plot. Here, the probability the model assigns to each sample to belong to the responder group, i.e., group “R,” is plotted. A large spread of the responder and non-responder data and a low amount of overlap are associated with a powerful prediction model. Figure 2B shows a strip plot of only one model among the models generated within the resampling, namely, the model whose score was closest to the mean score over the resampling iterations. The achieved performance of this model is summarised in Table 3. The final prediction model achieved a ROC AUC of 90% in the training cohort. The ROC AUC was slightly reduced in the validation cohort but was still at 82%.

**Table 3:** Prediction scores of the prediction model with ROC AUC score closest to the resampling mean ROC AUC score (see text for details)

Metric	Score
Accuracy	0.78
ROC AUC	0.90
Sensitivity	0.83
Specificity	0.66
PPV	0.83
NPV	0.67

### Validation of microarray data

**Fig. 3:**
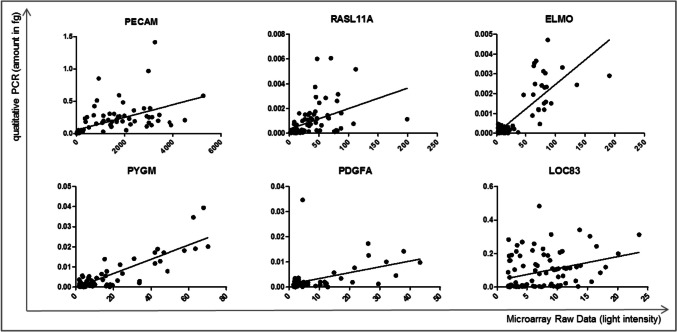
Validation of microarray data using quantitative PCR. Results obtained via quantitative PCR were correlated to raw microarray data for gene candidates identified as a robust signature for response prediction

To validate the microarray data, we selected genes identified as preliminary markers via microarray analysis (Supplementary Table 1) and performed quantitative PCR, including all samples—CD4 + and CD14 + . The quantitative PCR revealed that the gene expression is correlated with microarray data signal intensity (Fig. 3 and Supplementary Table 2).

## Discussion

In the PreTheraX study, we aimed to identify pharmacogenomic biomarkers to predict response to MTX in RA patients. We assessed gene expression signatures of CD14 + and CD4 + mononuclear cells isolated from peripheral blood samples of patients naïve to MTX, who were then administered MTX and followed up for 3 months. Our study revealed five distinct gene signatures—two in CD14 + and three in CD4 + cells—in responders versus non-responders. Our prediction model based on these led to a ROC AUC of 90% in our cohort, thus indicating an excellent predictive value. After applying a bootstrapping procedure to validate our findings within the same cohort, the model still achieved a ROC AUC of 82%. Interestingly, out of 70 patients finally available for analysis of response to MTX, only 15 were classified as non-responders.

Our study may have implications for future clinical practice if the results stand in validation studies. Identifying the five gene signatures can open the possibility to improve outcomes in clinical practice by developing personalized treatment plans. Those who are predicted to respond well to MTX would be recommended therapy with MTX as a first-line treatment option, while those who are less likely to respond could be considered for alternative therapies from the outset.

One other study assessed the role of monocytes in MTX response in RA: In 2014, Chara and colleagues simply looked at monocyte numbers [7]. In their cohort of 55 previously untreated RA patients, they found that the number of monocytes at baseline was significantly increased in MTX non-responders. The peripheral blood monocyte count remained elevated throughout the whole study period of six months. At a cut-off value of 650 cells/μl, the prediction model, which only divided patients by monocyte count, yielded an ROC AUC of 89%. A prediction model including only the count of a specific monocyte subset, namely, CD16 + monocytes with a high count of CD14 + , increased this ROC AUC further to 94%. However, this prediction model was not further validated.

Sergeant et al. assessed clinical and demographic characteristics only in the large Rheumatoid Arthritis Medication Study [12]. A total of 449 patients (43%) were classified as non-responders. In the multiple logistic regression model, rheumatoid factor negativity, higher HAQ scores, more tender joints, lower DAS28-CRP, and higher hospital anxiety and depression scale anxiety scores were associated with non-response. The model’s AUC ROC achieved an excellent score of 74%, which could be further improved by including biomarkers.

Genetic variants may also play a role in predicting response to MTX in RA: Single nucleotide polymorphisms (SNPs) connected to MTX pharmacokinetics, pharmacodynamics, and mechanisms of action were analysed in various studies. For example, genes coding for ATIC (aminoimidazole carboxamide ribonucleotide transformylase) [13–16], AMPD1 (adenosine monophosphate deaminase) [13, 15], and MTHFD1 (methylenetetrahydrofolate dehydrogenase) [13, 15] have come up repeatedly. In conjunction with clinical and demographic variables, the SNPs above had good predictive properties with final AUC ROC values in multivariable models of up to 84–85% in the training cohorts [13, 15, 16], which were reduced by about ten percentage points in most validation cohorts.

Hambardzumyan et al. investigated serum levels of 12 proteins in patients taking part in the SWEFOT trial. Early RA patients received MTX monotherapy for 3 months before being randomised to other treatments in the case of inadequate response [17]. After stepwise logistic regression analysis, four proteins were significantly associated with low DAS28 after 3 months: VCAM-1 (vascular cell adhesion molecule 1; odds ratio (OR; multivariable model) = 8.2), TNF-RI (tumour necrosis factor receptor I; OR = 2.5), CRP (OR = 0.99), and leptin (OR = 0.97). While no standard AUC ROC calculation was performed, the high ORs of VCAM-1 and TNF-RI might indicate good predictive properties, and serum protein levels might be easily measured in clinical practice (in contrast to genetic variants).

Apart from evaluating purely “human” biomarkers, it has been suggested that the gut microbiome might also play a role in the treatment response in RA. Artacho et al. included drug-naïve early RA patients and performed metagenomic sequencing of the gut microbiome. The abundance of several bacterial taxa was associated with later responses to treatment: The final ROC AUC of 84% indicated good discriminative performance. However, this study only assessed patients with oral MTX use, and the sample size was relatively small. While these results provide an interesting perspective on the potential role of gut microbiota in the treatment with oral MTX, the clinical implications might remain limited due to impracticability.

This study has several limitations. First of all, the sample size was only moderate. This made splitting the cohort into one training and one validation cohort impossible due to the lack of adequate statistical power. However, we performed a bootstrapping procedure to conduct validation analyses within our sample. Although this is no replacement for a proper validation, it confirmed our primary results. Furthermore, our study did not measure some previously identified markers predictive of MTX response, e.g., socioeconomic status and comorbidities. Moreover, joint ultrasound, which is a highly sensitive method to assess arthritis [18], was not performed. A strength is the prospective design of our study with preplanned measurements and analyses.

## Conclusion

In this cohort of MTX-naïve RA patients, genome-wide gene expression analysis of CD4 + and CD14 + cells predicted response to MTX at three months. Validation in an independent cohort is required to underpin our findings. Especially if used in conjunction with other predictive variables, these findings might facilitate the implementation of a prediction model for clinical practice to improve outcomes in RA.

### Supplementary Information

Below is the link to the electronic supplementary material.

Supplementary file1 (PDF 366 KB)

## Data Availability

The datasets analysed during the current study are available from the shared last authors on reasonable request.
