# Supplementary material for: Identification of gene expression biomarkers to predict clinical response to methotrexate in patients with rheumatoid arthritis
Source: Clin Rheumatol. 2023 Nov 17;43(1):511–9. doi: 10.1007/s10067-023-06814-2 (PMC10774206; doi:10.1007/s10067-023-06814-2)
Supplement: Supplementary file 1 — Supplementary file1 (PDF 366 KB) [file 10067_2023_6814_MOESM1_ESM.pdf]

Supplementary Tables

Cell type	Array Position	Gene ID	Gene Symbol	Gene Name
CD4	A_19_P00807468	lnc-HIST1H1A-1:2	lnc-HIST1H1A-1	lnc-HIST1H1A-1:2
	A_21_P0008131	lnc-CENPJ-2:1	lnc-CENPJ-2	lnc-CENPJ-2:1
	A_33_P3519424	lnc-CMPK2-2:2	lnc-CMPK2-2	lnc-CMPK2-2:2
	A_23_P140830	NM_024712	ELMO3	engulfment and cell motility 3
	A_23_P14124	NM_206827	RASL11A	RAS-like, family 11, member A
	A_23_P60793	NR_026710	ASMTL-AS1	ASMTL antisense RNA 1
	A_21_P0007921	ENST00000438640		
	A_23_P405815	NM_005609	PYGM	phosphorylase, glycogen, muscle
	A_23_P397391	NM_005306	FFAR2	free fatty acid receptor 2
	A_21_P0012018	ENST00000431506		ens|ENST00000431506|linc|TCONS_l2_00015431
	A_23_P4400	NM_033059	KRTAP4-11	keratin associated protein 4-11
	A_21_P0003526	ENST00000504957		ens|ENST00000504957|linc|lnc-FBXW7-1:1|linc|TCONS_00007882|gb|CR740783
	A_21_P0009578	lnc-FBXO15-4:1	lnc-FBXO15-4	lnc-FBXO15-4:1
	A_21_P0005593	lnc-PDGFA-2:1	lnc-PDGFA-2	lnc-PDGFA-2:1
	A_21_P0012255	XM_006726834	LOC102724248	uncharacterized LOC102724248
	A_21_P0003364	ENST00000508241		ens|ENST00000508241|linc|lnc-PDGFRA-2:1|linc|TCONS_00007531|tc|THC2658373
	A_21_P0014921	NR_002924	TBC1D3P1-DHX40P1	TBC1D3P1-DHX40P1 readthrough transcribed pseudogene
	A_21_P0006472	lnc-FLNA-1:1	lnc-FLNA-1	lnc-FLNA-1:1
	A_21_P0006871	XR_246208	LOC101927584	uncharacterized LOC101927584
	A_33_P3375348	Agilent Probe		
	A_33_P3229402	NM_000442	PECAM1	platelet/endothelial cell adhesion molecule 1
	A_24_P42389	ENST00000338352	OTUD6A	OTU deubiquitinase 6A
	A_21_P0006867	lnc-C10orf136-1:11	lnc-C10orf136-1	lnc-C10orf136-1:11
	A_19_P00322121	ENST00000454968	LINC00963	long intergenic non-protein coding RNA 963
	A_21_P0014136	BX369009		
	A_33_P3218960	NM_021098	CACNA1H	calcium channel, voltage-dependent, T type, alpha 1H subunit
	A_23_P391857	NM_004452	ESRRB	estrogen-related receptor beta
CD14	A_21_P0005772	lnc-CHRAC1-1:3	lnc-CHRAC1-1	lnc-CHRAC1-1:3
	A_33_P3305243	ENST00000373903	PSMD5	proteasome (prosome, macropain) 26S subunit, non-ATPase, 5
	A_19_P00807507	lnc-MRGPRF-4:2	lnc-MRGPRF-4	lnc-MRGPRF-4:2
	A_23_P420692	ENST00000295706	PPFIA4	protein tyrosine phosphatase, receptor type, f polypeptide (PTPRF), interacting protein (liprin), alpha 4
	A_33_P3321130	ENST00000520482	DENND3	DENN/MADD domain containing 3
	A_23_P252306	NM_002165	ID1	inhibitor of DNA binding 1, dominant negative helix-loop-helix protein
	A_21_P0004513	ENST00000514258	CRHBP	corticotropin releasing hormone binding protein

	A_23_P353667	NR_027148	MIR7-3HG	MIR7-3 host gene (non-protein coding)
	A_21_P0004219	NR_024424	LINC01018	long intergenic non-protein coding RNA 1018
	A_21_P0006199	lnc-ZNF462-1:3	lnc-ZNF462-1	lnc-ZNF462-1:3
	A_33_P3748714	NM_032664	FUT10	fucosyltransferase 10 (alpha (1,3) fucosyltransferase)
	A_21_P0008062	lnc-LRCH1-1:1	lnc-LRCH1-1	lnc-LRCH1-1:1
	A_33_P3389758	Agilent Probe		
	A_23_P164100	NM_181707	C17orf64	chromosome 17 open reading frame 64
	A_33_P3386262	NM_030928	CDT1	chromatin licensing and DNA replication factor 1
	A_24_P75190	NM_000519	HBD	hemoglobin, delta
	A_23_P203558	NM_000518	HBB	hemoglobin, beta
	A_21_P0000589	NM_001256795	LOC100129083	uncharacterized LOC100129083
	A_33_P3236628	ENST00000595085	BCKDHA	branched chain keto acid dehydrogenase E1, alpha polypeptide

Supplementary Table 1. Summary of differentially expressed genes in CD4+ and CD14+ cells in MTX responders and non-responders.

Gene name	PCR probe	product size	Primer forward	Primer reverse	SpearmanR	p value
**PECAM1**	ATCCCAATATGGAAGCTAACAGTCATTACG	101 bp	aaatgtccaggccagcagta	tgcatggtttctgacatcgt	0.8146	< 0.0001
**RASL11A**	CAT TTG TGC AGC TAA AAG ACT GGG CTT CTC	107 bp	ggggtgaactatctcagacagatg	ccc tataaaaataaactctgaatgtgtga	0.6404	< 0.0001
**ELMO3**	AAGCAGAACAAGGACCTCTATGAGTTGGCC	97 bp	tcctgacaggcaaggactg	cccacggtcatagctgattga	0.6187	< 0.0001
**PYGM**	TGTACAAGAACCCAAGAGAGTGGACGCGGA	91 bp	aatgccaggagaaagtcagc	gtgcggtcactggagaactt	0.5680	< 0.0001
**lnc-PDGFA-2**	AACTGGACCATGGAGGACGAGATGCATGTG	113 bp	gtttcttcggccttgctctc	cgtgtttggtgagttcggtt	0.4775	< 0.0001
**LOC83**	CAGTAGCCATAAGCTGGGGTAGACCCTGTC	150 bp	gtgctcatttccacctctcg	catctctgtgccctgctctt	0.3438	0.0006

Supplementary Table 2. Correlation of Microarray data and quantitative PCR results.
